# Marine-derived surfactin derivatives suppress collagen production and interfere with the HSP47-collagen interaction in preclinical models

**DOI:** 10.3389/fphar.2026.1809175

**Published:** 2026-04-28

**Authors:** Daisuke Okuno, Noriho Sakamoto, Yoshiko Akiyama, Chiaki Iketani, Ritsuko Murakami, Takuto Miyamura, Hirokazu Yura, Takashi Kido, Hiroshi Ishimoto, Yuji Ishimatsu, Hirokazu Taniguchi, Susumu Fukahori, Shinnosuke Takemoto, Takahiro Takazono, Tomoya Nishino, Jun Ishihara, Jun Takouda, Kohsuke Takeda, Yoshimasa Tanaka, Hiroshi Mukae

**Affiliations:** 1 Department of Respiratory Medicine, Nagasaki University Hospital, Nagasaki, Japan; 2 Department of Respiratory Medicine, Graduate School of Biomedical Sciences, Nagasaki University, Nagasaki, Japan; 3 Department of Nursing, Graduate School of Biomedical Sciences, Nagasaki University, Nagasaki, Japan; 4 Clinical Oncology Center, Nagasaki University Hospital, Nagasaki, Japan; 5 Department of Nephrology, Nagasaki University Graduate School of Biomedical Sciences, Nagasaki, Japan; 6 Department of Pharmaceutical Organic Chemistry, Nagasaki University Graduate School of Biomedical Sciences, Nagasaki, Japan; 7 Department of Cell Regulation, Nagasaki University Graduate School of Biomedical Sciences, Nagasaki, Japan; 8 Center for Medical Innovation, Nagasaki University, Nagasaki, Japan

**Keywords:** heat shock protein 47, Idiopathic pulmonary fibrosis, lung fibroblast, marine microoganisms, surfactin

## Abstract

**Introduction:**

Pulmonary fibrosis is a chronic and relentlessly progressive interstitial lung disease characterized by irreversible scarring of the lung parenchyma, for which no curative therapies currently exist. Heat shock protein 47 (HSP47), a collagen-specific molecular chaperone essential for proper collagen folding and secretion, has emerged as a compelling therapeutic target to mitigate pathological collagen deposition.

**Methods:**

To identify inhibitors of the HSP47–collagen interaction, we comprehensively screened a marine microorganism extract library. Initial screening revealed 16 extracts with inhibitory activity, and subsequent secondary screening narrowed these down to seven candidates. Among them, one extract exhibited potent, concentration-dependent inhibition of HSP47 activity and markedly suppressed collagen synthesis in lung fibroblasts at low concentrations. This lead extract was fractionated using high-performance liquid chromatography, and bioactive components were subsequently isolated.

**Results:**

Mass spectrometric analysis identified the active compounds as C14- and C15-surfactin, cyclic lipopeptides produced by *Bacillus subtilis*. Treatment with surfactin significantly attenuated collagen production in pulmonary fibroblasts without altering HSP47 expression, suggesting inhibition through disruption of the HSP47–collagen interaction. Furthermore, oral administration of surfactin markedly reduced lung hydroxyproline content, demonstrating substantial antifibrotic efficacy *in vivo*.

**Conclusion:**

Collectively, surfactin are novel inhibitors of the HSP47–collagen interaction with promising therapeutic potential for pulmonary fibrosis treatment.

## 1 Introduction

Idiopathic pulmonary fibrosis (IPF) is a specific form of chronic, progressive fibrosing interstitial pneumonia of unknown cause, characterized by worsening dyspnea, decline in lung function, and a usual interstitial pneumonia pattern, with a poor prognosis (Lederer and Martinez, 2018; Raghu et al., 2018). The fibrotic process is driven by recurrent epithelial cell injury, senescence of alveolar epithelial cells, and the release of pro-fibrotic mediators, which collectively activate fibroblasts and myofibroblasts to synthesize and deposit extracellular matrix (ECM) components (Lederer and Martinez, 2018). Among these, type I collagen constitutes the predominant ECM protein, accumulating excessively under chronic fibrotic conditions (Kai et al., 2016).

Heat shock protein 47 (HSP47) is a stress-inducible collagen-binding molecular chaperone that facilitates procollagen maturation within the endoplasmic reticulum (ER) (Bellaye et al., 2021). Elevated HSP47 expression has been documented in type II alveolar epithelial cells and fibroblasts from patients with IPF, as well as in multiple experimental models of fibrosis, implicating HSP47 as a crucial mediator of fibrogenesis (Iwashita et al., 2000; Ishii et al., 2003; Nishino et al., 2003; Kakugawa et al., 2004). In hepatic and pancreatic fibrosis models, small interfering RNA-mediated silencing of HSP47 markedly reduced collagen secretion and effectively attenuated disease progression (Sato et al., 2008; Ishiwatari et al., 2013). Current pharmacologic interventions for IPF, including nintedanib and pirfenidone, modestly slow disease progression and improve patient outcomes; however, they fail to reverse established fibrosis (King et al., 2014; Raghu et al., 2022; Richeldi et al., 2014). Consequently, the development of novel therapeutic strategies remains an urgent priority, and numerous investigational agents are under clinical evaluation (Cruwys et al., 2024). Based on accumulating evidence, HSP47 has emerged as a promising therapeutic target for antifibrotic intervention.

In the search for novel antifibrotic agents targeting HSP47, naturally derived compounds have attracted considerable attention. Surfactants can also be naturally produced by microorganisms, such as bacteria, yeasts, and fungi, and these microbially derived compounds are collectively termed biosurfactants (Sharma et al., 2021). Biosurfactants have attracted attention as potential therapeutic agents owing to their diverse biological activities, including antioxidant, antimicrobial, anti-inflammatory, and anticancer properties (Kumar et al., 2021; Al-Kashef et al., 2023). Among these, surfactin is one of the most extensively studied biosurfactants and reportedly exerts multiple biological effects, including anti-apoptotic, anti-cancer, and anti-inflammatory activities (Zhang et al., 2015; Wu et al., 2017; Vo et al., 2020). In the context of fibrosis, surfactin produced by B. halotolerans—a cyclic lipopeptide with an m/z of 994.20, comprising a C12 fatty acid side chain and seven amino acids—was previously shown to attenuate bleomycin-induced pulmonary fibrosis (Mousa et al., 2024). This antifibrotic effect was primarily attributed to the inhibition of extracellular matrix production via suppression of the TGF-β1/Smad3 signaling pathway. Additionally, surfactin has been shown to reduce NF-κB activation, tumor necrosis factor-α expression, and CD68^+^ macrophage infiltration, thereby exerting anti-inflammatory effects that further contribute to fibrosis attenuation.

Previously, we established a screening platform for HSP47 inhibitors and identified potential candidates from our proprietary compound library (Okuno et al., 2021). A critical aspect of drug development is the discovery of novel structural scaffolds for small- and mid-sized biomolecules, as these unique frameworks often serve as the foundation for innovative therapeutic agents. To achieve this, libraries encompassing diverse chemical space and molecular weights are indispensable (Dobson, 2004; Chen et al., 2018). Marine microorganisms have garnered considerable attention as a source of novel bioactive compounds. Considering that most genetic and metabolic diversity resides within microorganisms (Sogin et al., 2006; Ahmad et al., 2019), marine-derived natural products exhibit broad biological activities through interactions with diverse biomolecular targets, including enzymes and receptors (Calixto, 2019). Marine microorganisms produce a wide spectrum of biomolecules, ranging from small molecules to mid-sized cyclic peptides. Thus, leveraging an original marine microorganism extract library is a rational strategy for the discovery of novel lead compounds and therapeutic agents (Bugni et al., 2008; Glaser and Mayer, 2009). In this study, we utilized our marine microorganism extract library to screen for inhibitors of the HSP-collagen interaction to identify new drug candidates for the treatment of pulmonary fibrosis.

## 2 Materials and methods

### 2.1 HSP47 inhibitor screening

Overall, 480 marine microorganism extracts were screened for HSP47 inhibitory activity using a previously established protocol (Thomson and Ananthanarayanan, 2000; Okuno et al., 2021). Human HSP47 (1.34 μM), type I collagen (0.67 μM), and test extracts (500 μg/mL) were combined in 96-well round-bottom plates. The mixtures were transferred to ultraviolet (UV)-transparent, flat-bottom 96-well microplates, and collagen fibril formation was monitored at 313 nm for 80 min at 34 °C using a PHERAstar multimode plate reader. The percentage of fibril formation was calculated as follows (Thomson et al., 2005): Percent fibril formation = [(ΔA–μ^−^)/(μ^+^ –μ^-^)] × 100, where ΔA represents the change in optical density from 0 to 80 min, μ^−^ is the mean ΔA of negative control wells (without extracts), and μ^+^ is the mean ΔA of positive control wells (collagen only). Marine microorganism extracts inducing ≥20% fibril formation, corresponding to values exceeding 3 standard deviations above the mean of the negative controls (Thomson et al., 2005), were considered positive, and those consistently positive in two independent assays were classified as hits. Dose-dependent inhibitory effects of the hit extracts were subsequently confirmed using a concentration-gradient fibril formation assay.

### 2.2 Human and mouse lung fibroblast culture

Diseased human lung fibroblasts (DHLF) derived from a patient with IPF (Lonza Walkersville, Inc., Basel, Switzerland) and mouse lung fibroblasts (Mlg; American Type Culture Collection, Manassas, VA, United States) were cultured in complete RPMI1640 medium (Merck & Co., Inc., Darmstadt, Germany) supplemented with 10% fetal calf serum (Merck & Co., Inc.), 10^–5^ M of 2-mercaptoethanol (Wako Pure Chemical Industries, Ltd., Osaka, Japan), 100 μg/mL streptomycin (Meiji Seika Pharma Co., Ltd., Tokyo, Japan), and 100 U/mL penicillin (Meiji Seika Pharma Co., Ltd.) in 75-cm^2^ flasks (Corning Inc., Corning, NY, United States) at 37 °C in a humidified atmosphere containing 5% CO_2_. Cells between passages 3 and 7 were used for all experiments.

### 2.3 Administration of experimental agents to lung fibroblasts

DHLF cells (1 × 10^5^ cells/well) or Mlg cells (1.5 × 10^5^ cells/well) were seeded into six-well plates and cultured overnight. Cells were subsequently treated with either marine extract (12.5 or 25 μg/mL), surfactin (various concentrations; 10–100 μM), or vehicle control. After 2 d of incubation, protein expression levels of collagen, HSP47, fibronectin, and alpha-smooth muscle actin (α-SMA) were analyzed by Western blotting.

### 2.4 Western blot analysis

Immunoblotting was performed as previously described (Okuno et al., 2022). Primary antibodies included rabbit anti-type I collagen polyclonal antibody (1:5000; Thermo Fisher Scientific, Waltham, MA, United States), rabbit anti-fibronectin polyclonal antibody (1:1000; Abcam, Cambridge, UK), mouse anti-αSMA antibody (1:1000; Abcam), rabbit anti-SERPINH1 polyclonal antibody (1:1000; Invitrogen, Thermo Fisher Scientific), and mouse anti-glyceraldehyde 3-phosphate dehydrogenase (GAPDH) polyclonal antibody (1:1000; Thermo Fisher Scientific). Secondary antibodies were horseradish peroxidase-labeled anti-rabbit immunoglobulin G (IgG) antibody (1:5000; Abcam) and anti-mouse IgG antibody (1:1000; Bio-Techne, Minneapolis, MN, United States). Relative protein expression was quantified by normalizing to GAPDH using Image Lab™ software ver. 5.2.1 (Bio-Rad Laboratories, Hercules, CA, United States).

### 2.5 Luminescence-based cytotoxicity assay

DHLF cells (2 × 10^4^ cells) were seeded into 96-well flat-bottom plates (Corning Inc.) and cultured overnight. Cells were subsequently treated with the hit marine microorganism extract at concentrations of 0, 1, 3, 10, 30, and 100 μg/mL. After 24 h of incubation, wells were washed twice, and 100 μL of CellTiter-Glo® Reagent (PerkinElmer Inc., Waltham, MA, United States) was added. Cell lysates were transferred to 96-well OptiPlates (PerkinElmer Inc.), and luminescence was measured using a NIVO multi-plate reader (PerkinElmer Inc.). All experiments were performed in triplicate.

### 2.6 High-performance liquid chromatography (HPLC)

HPLC analysis was performed using a Shimadzu system (Shimadzu Corp., Kyoto, Japan) comprising a system controller (SCL-10A), solvent delivery unit (LC-10AD), UV–visible detector (SPD-10Avp), autosampler (SIL-10ADvp), and online degasser (DGU-14A). Separation was achieved on a Triart Phenyl column (5 µm particle size, 12 nm pore size, 4.6 × 250 mm; YMC Co., Ltd., Kyoto, Japan). The column temperature was maintained at 40 °C using a column oven (Omron model E5C3; Omron, Tokyo, Japan). The mobile phase consisted of solvent A (water containing 0.1% trifluoroacetic acid [TFA]) and solvent B (acetonitrile with 0.1% TFA). The flow rate was set at 1.0 mL/min, and detection was performed at 210 nm. Samples were prepared at a concentration of 10 mg/mL in acetonitrile, and 20 μL were injected. Eluted fractions were collected and evaporated for 3 h using a centrifugal concentration system comprising a microcentrifuge (CC-105; TOMY Seiko Co., Ltd., Tokyo, Japan), vacuum concentrator (VA-500F; TAITEC Corporation, Saitama, Japan), and rotary vacuum pump (GLD-051; ULVAC, Inc., Kanagawa, Japan). Dried samples were reconstituted into 10 μL of dimethylsulfoxide for subsequent experiments and analysis.

### 2.7 Mass spectrometry analysis

Mass spectrometric analysis of active fractions from marine microorganism extracts (fractions 8 and 10) and surfactin fractions (fractions 4 and 5) was performed using a matrix-assisted laser desorption/ionization time-of-flight (MALDI-TOF) mass spectrometer (Ultraflex III, Bruker Daltonics, Bremen, Germany). Samples collected after HPLC fractionation were dried and reconstituted in acetonitrile/water (70:30). For each measurement, 0.5 μL of the sample was mixed with 0.5 μL of matrix solution (α-cyano-4-hydroxycinnamic acid, 0.7 mg/mL) on the target plate. Mass spectra were acquired over an m/z range of 0–2000. Data were processed and analyzed using FlexAnalysis software (Bruker Daltonics).

### 2.8 Immunocytochemistry

DHLF cells (2.5 × 10^4^ cells) were seeded into Lab-Tek chamber slides (Thermo Fisher Scientific) and cultured overnight. After removing the supernatant, cells were treated with surfactin (30 μM) or vehicle and incubated for an additional 2 d. Type I collagen expression was then visualized by immunocytochemistry, as previously described (Okuno et al., 2022). An anti-collagen type I polyclonal antibody (Thermo Fisher Scientific) was used, with normal rabbit IgG (200 μL, Santa Cruz Biotechnology Inc., Santa Cruz, CA) serving as the negative control.

### 2.9 Mouse experiments

C57BL/6J 8-week-old mice (The Jackson Laboratory Japan, Inc., Yokohama, Japan) were used. Mice received either bleomycin (1.5 mg/kg; Nippon Kayaku Co., Ltd., Tokyo, Japan) or sterile saline (70 µL) by intratracheal instillation (Moore and Hogaboam, 2008). Beginning the following day, mice were administered surfactin sodium salt (200 mg/kg; FUJIFILM Wako Pure Chemical Corporation) or vehicle (phosphate-buffered saline [PBS], 200 µL) by daily oral gavage from day 1–20 (Mousa et al., 2024). On day 21, mice were euthanized, and lung tissues were harvested. Bronchoalveolar lavage (BAL) was performed by administering 1 mL of PBS twice via the trachea. Recovered BAL fluid (18 µL) was mixed with AO/PI reagent (2 μL; Logos Biosystems, Inc., Anyang, Republic of Korea) and loaded onto a LUNA Cell Count Slide (Logos Biosystems, Inc.). Cell numbers were determined using a LUNA Cell Counter (Logos Biosystems, Inc.). BAL fluid was subsequently centrifuged at 400 *g* for 5 min at 4 °C, and the supernatant was collected and stored at −80 °C until analysis. Protein concentrations in thawed BAL supernatants were determined using a bicinchoninic acid assay kit (Thermo Fisher Scientific) according to the manufacturer’s instructions. The left lung was fixed in formalin (Japan Tanner Co., Ltd., Osaka, Japan) for histological analysis, while the right lung was homogenized in saline and stored at −80 °C for subsequent assays.

### 2.10 Collagen quantification

Hydroxyproline content in homogenized right lungs was measured using a hydroxyproline assay kit (QuickZyme Biosciences B.V., Leiden, Netherlands) according to the manufacturer’s instructions. Hydroxyproline concentrations (µM) were normalized to right lung weight and expressed as µM/g for group comparisons.

### 2.11 Masson’s trichrome staining

Left lung sections were subjected to Masson’s trichrome staining according to the manufacturer’s instructions. Images were acquired using a microscope (BZ-X710; KEYENCE Corporation, Osaka, Japan) at 10× magnification.

### 2.12 Statistical analysis

Statistical analyses were performed using one-way analysis of variance followed by Tukey’s or Dunnett’s multiple comparison test, or Student’s *t*-test, in GraphPad Prism (version 9.4.0; GraphPad Software, CA, United States). Statistical significance was set as p < 0.05.

## 3 Results

### 3.1 Screening of a marine microorganism extract library to identify HSP47 inhibitors

**FIGURE 1:**
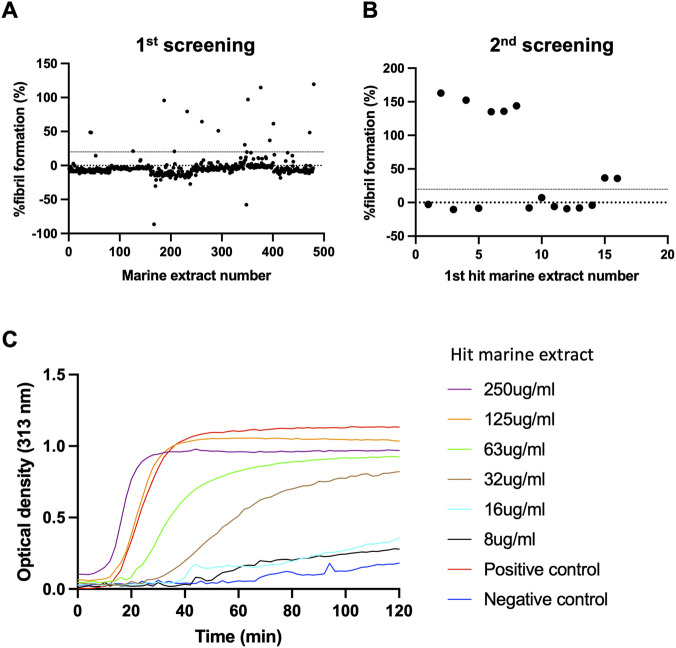
Screening of a marine microorganism extract library to identify inhibitors of the collagen-HSP47 interaction. **(A)** Primary screening for HSP47 inhibitors. Overall, 480 marine microorganism extracts (500 μg/mL) were tested by incubating HSP47 (1.34 μM) and type I collagen (0.67 μM), followed by monitoring fibril formation at 313 nm for 80 min. Fibril formation was used as the primary readout in this assay. Extracts exhibiting ≥20% fibril formation were considered positive. **(B)** Secondary screening of extracts identified in the primary screen under identical conditions. Seven extracts were confirmed as positive hits. **(C)** Dose-dependent inhibition of HSP47 activity by a representative hit extract at a concentration lower than those used in the initial screening. HSP47, heat shock protein 47.

**FIGURE 2:**
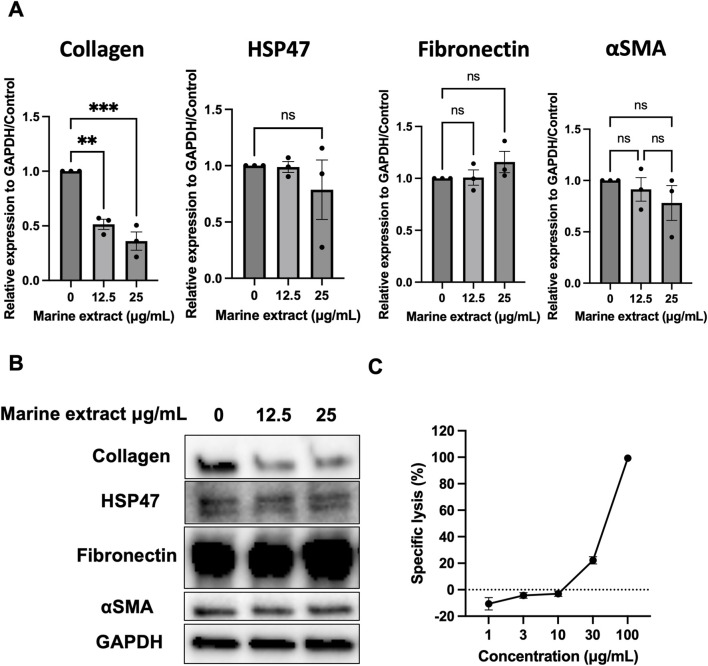
Inhibition of collagen expression in lung fibroblasts by a hit marine extract. **(A,B)** Western blot analysis of type I collagen, HSP47, fibronectin, and α-SMA in lung fibroblasts treated with a representative hit marine microorganism extract. DHLF cells (1 × 10^5^) were cultured overnight in six-well plates and then treated with the extract (12.5 or 25 μg/mL) or vehicle control. After 2 d, protein levels were assessed by Western blotting. Relative collagen expression was normalized to GAPDH. Data represent mean ± standard deviation from three independent experiments. Statistical significance was determined by one-way ANOVA with multiple comparisons (***p < 0.001, **p < 0.01). **(C)** Cell viability of fibroblasts treated with the hit marine microorganism extract. DHLF cells (2 × 10^4^) were cultured overnight and then treated with the extract at concentrations of 0, 1, 3, 10, 30, and 100 μg/mL. After 24 h, cell viability was assessed by measuring ATP levels using a standard luciferase-based assay. α-SMA, α-smooth muscle actin; HSP47, heat shock protein 47; ANOVA, analysis of variance; GAPDH, glyceraldehyde 3-phosphate dehydrogenase; ATP, adenosine triphosphate; DHLF, diseased human lung fibroblasts.

To identify compounds that inhibit human HSP47, we screened marine microorganism extracts using a previously established assay for identifying HSP47 (Okuno et al., 2021). In the primary screening, extracts from 480 marine microorganisms (500 μg/mL) were tested by incubating HSP47 (1.34 μM), type I collagen (0.67 μM), followed by monitoring fibril formation at 313 nm for 80 min. The percentage of fibril formation was calculated, and extracts exhibiting ≥20% fibril formation were considered positive and subjected to secondary screening (Figure 1A). Sixteen extracts met this criterion. In the secondary screening, conducted under identical conditions, seven extracts again demonstrated ≥20% fibril formation and were defined as confirmed hits (Figure 1B). Among these, one marine microorganism extract derived from *Bacillus subtilis* exhibited dose-dependent inhibition of HSP47 activity (Figure 1C) and was further evaluated for antifibrotic activity in human lung fibroblasts. Fibroblasts were treated with this extract then underwent Western blotting, revealing a dose-dependent suppression of collagen expression (Figures 2A,B). In contrast, no significant changes were observed in α-SMA or fibronectin expression, and HSP47 levels remained unchanged. Cytotoxicity was assessed using a luciferase-based viability assay in fibroblasts treated with the hit extract (Figure 2C). Cytotoxicity was detected at concentrations above 30 μM; however, collagen suppression was observed at non-cytotoxic concentrations.

### 3.2 Identification of active fractions from the hit marine extract that inhibited HSP47

**FIGURE 3:**
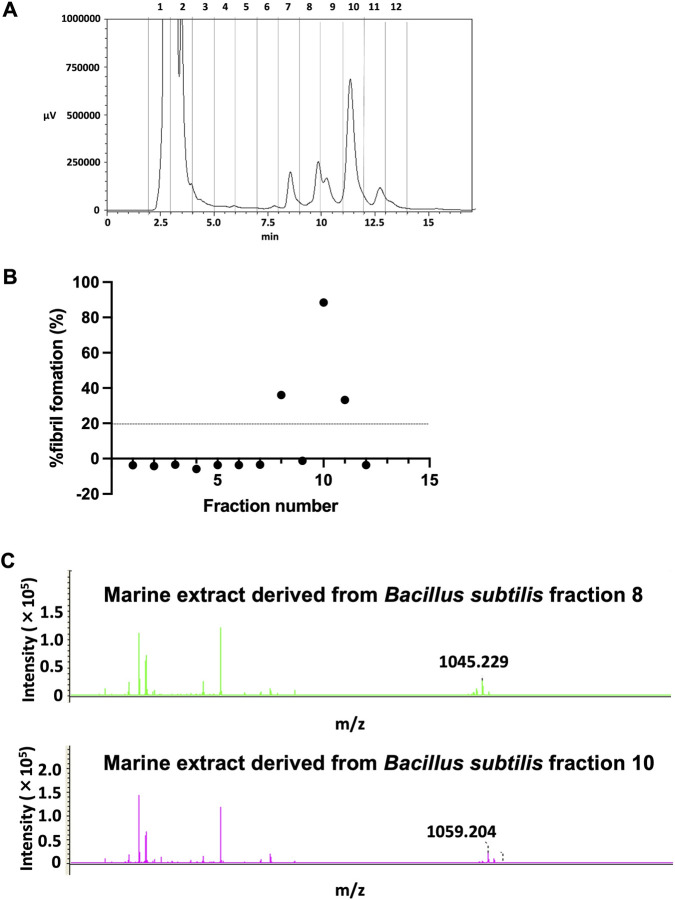
Identification of active fractions in the hit marine microorganism extract derived from *Bacillus subtilis* that inhibited HSP47. **(A)** Fractionation of the hit marine microorganism extract derived from *Bacillus subtilis* using HPLC. Precisely 20 µL of sample (10 mg/mL in acetonitrile) was injected onto a Triart Phenyl column (5 μm particle size, 12 nm pore size, 4.6 × 250 mm) maintained at 40 °C. Fractions were collected according to the chromatographic profile, evaporated for 3 h, and reconstituted in 10 μL of DMSO. **(B)** Screening of individual fractions for HSP47 inhibition. Each fraction (4 μL) was incubated with HSP47 (2.01 μM) and type I collagen (0.67 μM), and fibril formation was monitored at 313 nm for 80 min. Fractions exhibiting ≥20% inhibition were considered positive. **(C)** MALDI-TOF mass spectrometric analysis of two positive fractions. Distinct mass-to-charge (m/z) values were detected for each fraction, indicating the presence of two bioactive compounds. HSP47, heat shock protein 47; HPLC, high-performance liquid chromatography; MALDI-TOF, matrix-assisted laser desorption/ionization time-of-flight; DMSO, dimethylsulfoxide.

To identify the components responsible for inhibiting HSP47–collagen interactions and collagen production, the bioactive marine microorganism extract was fractionated using HPLC (Figure 3A). Each fraction was collected and subjected to the HSP47 screening assay under the same conditions described above, and the percentage of fibril formation was calculated. Three fractions (fractions 8, 10, and 11) exhibited inhibitory activity against HSP47 (Figure 3B). Comparison with the HPLC chromatogram suggested that the major bioactive constituents were present in fractions 8 and 10. These fractions were further analyzed by MALDI-TOF mass spectrometry to determine their molecular masses. The analysis revealed molecular ions at m/z 1045 for fraction 8 and m/z 1059 for fraction 10 (Figure 3C). Considering that the extract originated from *B. subtilis* and based on the m/z data, these active components were predicted to be surfactin derivatives produced by *B*. *subtilis*.

### 3.3 Identification of surfactin as the active HSP47-Inhibitory component

**FIGURE 4:**
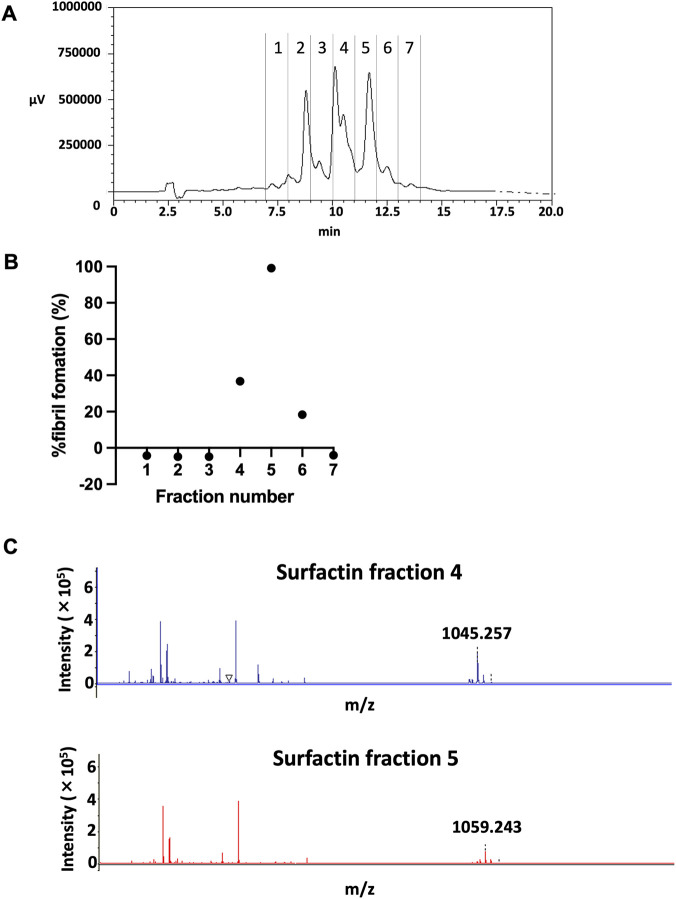
Characterization of HSP47-inhibitory fractions within commercially available surfactin. **(A)** Fractionation of commercially sourced surfactin using HPLC. A 20 μL aliquot of the surfactin (10 mM in water) was applied to a Triart Phenyl column maintained at 40 °C. Eluate fractions were collected according to the chromatographic profile, concentrated by rotary evaporation for 3 h, and subsequently reconstituted in 10 μL of DMSO. **(B)** Screening of individual fractions for HSP47 inhibition. Aliquots of each fraction (4 μL) were incubated with HSP47 (2.01 μM) and type I collagen (0.67 μM), and fibril formation was monitored spectrophotometrically at 313 nm for 80 min. Fractions exhibiting ≥20% fibril formation were classified as positive. **(C)** MALDI-TOF mass spectrometric analysis of two positive fractions. Both fractions displayed m/z signatures identical to those observed in the bioactive components of the hit marine extract, confirming the presence of two common inhibitory species. HSP47, heat shock protein 47; HPLC, high-performance liquid chromatography; MALDI-TOF, matrix-assisted laser desorption/ionization time-of-flight.

To confirm the identity of the bioactive components, commercially available surfactin was fractionated using HPLC, and individual fractions were collected according to their chromatographic patterns (Figure 4A). Each fraction was then tested in the HSP47 inhibition assay under identical conditions. Fractions 4 and 5 exhibited significant inhibitory activity against HSP47 (Figure 4B). Subsequent MALDI-TOF mass spectrometric analysis revealed molecular ions of m/z 1045 in fraction 4 and m/z 1059 in fraction 5 (Figure 4C). These values were identical to those obtained from the marine microorganism extract fractions 8 and 10, respectively, indicating that the active compounds were surfactin derivatives. Based on molecular ion information, fraction 4 was assigned as C14-surfactin and fraction 5 as C15-surfactin. Collectively, C14- and C15-surfactin were identified as the HSP47-inhibitory components present in the hit marine extract.

### 3.4 Antifibrotic activity of surfactin in human lung fibroblasts

**FIGURE 5:**
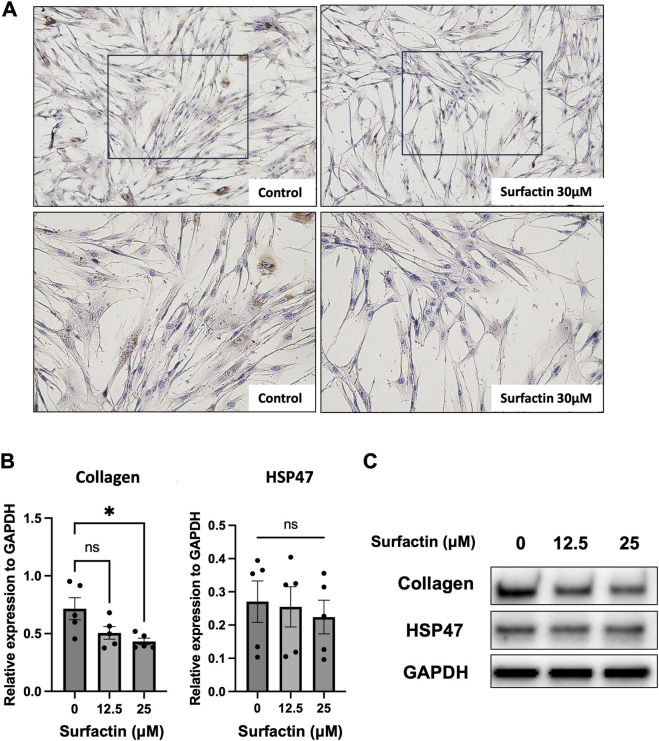
Suppression of type I collagen expression in lung fibroblasts by surfactin. **(A)** Immunocytochemical analysis of type I collagen in lung fibroblasts treated with surfactin. DHLF cells (2.5 × 10^4^) were seeded into Lab-Tek chamber slides and cultured overnight. After removing the supernatant, surfactin (30 μM) was added, and cells were incubated for an additional 2 d. Type I collagen expression was visualized by immunocytochemistry. **(B,C)** Western blot analysis of type I collagen and HSP47 in lung fibroblasts exposed to surfactin. DHLF cells (1 × 10^5^) were seeded into six-well plates, cultured overnight, and treated with surfactin (12.5 or 25 μM) or vehicle control for 2 d. Protein levels of type I collagen and HSP47 were assessed by Western blotting. Relative collagen expression was normalized to GAPDH. Data represent mean ± standard deviation from five independent experiments. Statistical significance was determined using one-way ANOVA with multiple comparisons (*p < 0.05). HSP47, heat shock protein 47; ANOVA, analysis of variance; DHLF, diseased human lung fibroblasts.

Subsequently, we evaluated the antifibrotic effects of surfactin in cultured human lung fibroblasts. Cells were treated with varying concentrations of surfactin and incubated for 48 h. Immunocytochemical analysis of type I collagen revealed a marked reduction in collagen deposition in surfactin-treated cells compared with controls (Figure 5A). Consistently, Western blot analysis confirmed a dose-dependent decrease in intracellular collagen expression without significantly affecting the expression of α-SMA or fibronectin (Figures 5B,C). In contrast, surfactin did not alter HSP47 protein levels, aligning with the observations obtained using the crude marine microorganism extract.

### 3.5 Antifibrotic effects of surfactin in preclinical models

**FIGURE 6:**
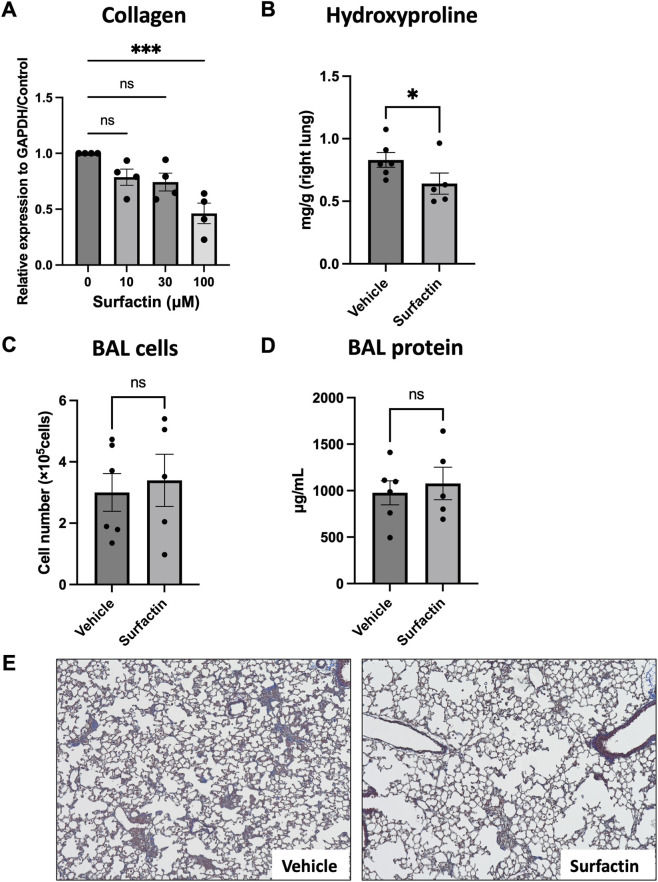
Antifibrotic properties of surfactin in mouse lung fibrotic models. **(A)** Western blot analysis of type I collagen in mouse lung fibroblasts treated with surfactin. Mlg cells (1.5 × 10^5^) were seeded into six-well plates, cultured overnight, and treated with surfactin at concentrations of 10, 30, or 100 μM or with vehicle control for 2 d. Type I collagen expression was analyzed by Western blotting and normalized to GAPDH. Data represent mean ± standard deviation from four independent experiments. Statistical significance was determined using one-way ANOVA with multiple comparisons (***p < 0.001). **(B)** Quantification of lung hydroxyproline content. C57BL/6 mice received intratracheal bleomycin (1.5 mg/kg). Beginning the following day, mice were administered surfactin (200 mg/kg) or vehicle by daily oral gavage. Mice were euthanized on day 21 for the collection of BAL and lung tissue. The right lungs were homogenized, and hydroxyproline content was determined and normalized to lung weight (mg/g). **(C,D)** BAL cell counts and total protein concentration. BAL fluid was centrifuged, and supernatants were analyzed for protein concentration using the BCA assay. Cell pellets were resuspended in PBS for total cell counting. Data represent mean ± standard deviation. Statistical significance was determined using the Student’s t-test (*p < 0.05). **(E)** Histological assessment of the left lungs by Masson’s trichrome staining. Representative images in each group are shown. GAPDH, glyceraldehyde 3-phosphate dehydrogenase; PBS, phosphate-buffered saline; BCA, bicinchoninic acid assay; BAL, bronchoalveolar lavage; Mlg, mouse lung fibroblasts.

To further assess the antifibrotic potential of surfactin, we examined the effects in murine systems. Mouse lung fibroblasts treated with surfactin for 48 h exhibited a significant, dose-dependent reduction in collagen expression, as determined by Western blotting (Figure 6A). To evaluate its therapeutic efficacy *in vivo*, we utilized a bleomycin-induced pulmonary fibrosis model. Mice received intratracheal bleomycin (1.5 mg/kg), followed by daily oral administration of surfactin (200 mg/kg) or vehicle. On day 21, BAL and lung tissue samples were collected. Hydroxyproline quantification in homogenized right lungs revealed a significant reduction in collagen content in surfactin-treated mice compared with that of controls (Figure 6B). BAL fluid analysis showed no significant differences in total cell counts or protein concentrations between groups (Figures 6C,D). Histological assessment of left lung sections by Masson’s trichrome staining demonstrated modest attenuation of fibrotic lesions in surfactin-treated mice (Figure 6E).

## 4 Discussion

IPF is a progressive and intractable disease wherefor no curative treatment currently exists, underscoring the urgent need for novel therapeutic strategies. HSP47 is instrumental suppress fibrotic progression, positioning HSP47 inhibitors as promising therapeutic candidates for pulmonary fibrosis.

Nagasaki University has a unique marine microorganism extract library comprising over 20,000 strains, encompassing a diverse array of small-to mid-sized biomolecules that modulate intracellular protein–protein interactions. Marine-derived biomolecules have long been recognized as valuable sources for drug discovery across various diseases (Tanaka et al., 2022). In this study, we screened this library to identify HSP47 inhibitors and successfully discovered two compounds—C14- and C15-surfactin—derived from *B*. *subtilis* as novel HSP47 inhibitors.

Pulmonary surfactant, a complex mixture of phospholipids and specific proteins secreted by alveolar type II epithelial cells, primarily functions to reduce alveolar surface tension, thereby maintaining alveolar stability. Additionally, it exhibits antioxidant and anti-inflammatory properties. The therapeutic potential of exogenously administered surfactants has been explored in various pulmonary disorders, including pneumonia, acute lung injury, and asthma (Han and Mallampalli, 2015). Experimental studies using pulmonary fibrosis models have demonstrated that exogenous surfactant treatment can prevent ECM deposition (Beike et al., 2019). Surfactin, a well-characterized biosurfactant, has been reported to attenuate pulmonary fibrosis in experimental models. Its antifibrotic effects are associated with suppression of extracellular matrix production and modulation of TGF-β–related and inflammatory signaling pathways.

In this study, we screened a marine microorganism extract library to identify biomolecules capable of inhibiting the interaction between HSP47 and type I collagen—a critical step in collagen maturation and fibrosis. This screening identified an extract exhibiting potent HSP47 inhibitory activity, which was subsequently fractionated using HPLC. Secondary screening of individual fractions revealed distinct bioactive components. Microbial identification and mass spectrometric analysis determined that these bioactive compounds were C14- and C15-surfactin produced by *B*. *subtilis*. Notably, the surfactin fraction presumed to be C13 did not exhibit HSP47 inhibitory activity, suggesting that inhibition may be specific to C14- and C15-surfactin.

Despite structural differences from previously reported surfactins, these biosurfactins similarly suppressed collagen deposition in a bleomycin-induced pulmonary fibrosis mouse model. In our study, commercially available surfactin was used to ensure consistent quality, purity, and reproducibility across experiments. The use of purified commercial surfactin enabled a more precise evaluation of its biological effects by minimizing variability associated with extraction and coexisting components. Surfactin suppressed collagen production in lung fibroblasts at non-cytotoxic concentrations without altering HSP47 expression, indicating that its antifibrotic effect results from disruption of the HSP47–collagen interaction rather than transcriptional regulation of HSP47. Surfactin did not significantly alter the expression of α-SMA or fibronectin. This selective effect on collagen may reflect the role of HSP47 as a collagen-specific molecular chaperone. Consistent with these *in vitro* findings, oral administration of surfactin in the bleomycin-induced pulmonary fibrosis model significantly reduced hydroxyproline content and improved histological features of fibrosis. Interestingly, BAL fluid analysis revealed no significant differences in total cell counts or protein concentrations between surfactin-treated and vehicle groups, suggesting that the antifibrotic effect was not primarily mediated by an anti-inflammatory mechanism.

To the best of our knowledge, this is the first report identifying surfactin as an inhibitor of HSP47-mediated collagen processing, providing novel mechanistic insight and highlighting surfactin as promising therapeutic candidates for pulmonary fibrosis.

This study has several limitations. First, although we identified C14- and C15-surfactin as active components by mass spectrometry, most functional analyses were performed using commercially available surfactin, which contains a mixture of isoforms. Therefore, it remains unclear whether the observed anti-fibrotic effects are attributable to individual surfactin isoforms or to potential synergistic effects among multiple components.

Second, due to technical limitations in isolating and quantifying sufficient amounts of individual isoforms from the marine-derived extract, we were unable to evaluate the *in vitro* and *in vivo* effects of purified C14- or C15-surfactin derived directly from the original extract.

Third, although our data suggest that surfactin interferes with the interaction between HSP47 and collagen, the precise molecular mechanism, including direct binding to HSP47, has not yet been fully elucidated. In addition, although we attempted to evaluate direct binding between surfactin and HSP47 using surface plasmon resonance, reliable assessment was technically challenging due to the physicochemical properties of surfactin, including its amphipathic nature, and we were unable to obtain conclusive evidence of direct interaction. Further studies are needed to clarify these points.

In conclusion, through screening of a marine microorganism extract library, we identified C14-and C15-surfactin, biosurfactants produced by *B*. *subtilis,* as novel inhibitors of HSP47. Surfactin suppressed fibrosis both *in vitro* and *in vivo* by disrupting the HSP47–collagen interaction, independently of anti-inflammatory mechanisms. Considering the absence of curative therapies for pulmonary fibrosis, these findings highlight surfactin as promising and innovative candidates for the development of novel antifibrotic therapeutics.

## Data Availability

The original contributions presented in the study are included in the article/supplementary material, further inquiries can be directed to the corresponding author.
